# Electro-Mechanical Coupling Analysis of L-Shaped Three-Dimensional Braided Piezoelectric Composites Vibration Energy Harvester

**DOI:** 10.3390/ma17122858

**Published:** 2024-06-11

**Authors:** Mengfei Sun, Ming Song, Gaofeng Wei, Fengfeng Hua

**Affiliations:** 1 School of Mechanical Engineering, Qilu University of Technology, Shandong Academy of Sciences, Jinan 250353, China; 10431210089@stu.qlu.edu.cn (M.S.); wgf@qlu.edu.cn (G.W.); 2 Shandong Institute of Mechanical Design and Research, Jinan 250353, China; 3 SDEE Hitachi High-Voltage Switchgear Co., Ltd., Xiamen 361101, China; hua-fengfeng@lnaeps.com

**Keywords:** L-shaped structure, 3D braided composites, energy harvester, piezoelectric effect, electro-mechanical coupling

## Abstract

In this article, an L-shaped three-dimensional (3D) braided piezoelectric composite energy harvester (BPCEH) is established, which consists of an elastic layer composed of a 3D braided composite, flanked by upper and lower layers of piezoelectric material and two tuning mass blocks. Glass fiber and epoxy resin are used to produce a 3D braided composite. This L-shaped 3D BPCEH is mechanically designable and can be adapted to different work requirements by varying the braided angle of the 3D braided composite layer. The material parameters of 3D braided composites are predicted for different braided angles by means of a representative volume element (RVE). Electro-mechanical coupled vibration equations for the L-shaped 3D BPCEH are established. The impact of braided angles on voltage and power output is discussed in this article. Simulations using finite element method are conducted to analyze the voltage and power output responses at various braided angles. In addition, the effects of the mass of mass block B and the length of the beam on the output performance of the L-shaped 3D BPCEH are analyzed.

## 1. Introduction

The development and utilization of new energy sources have garnered attention due to the widespread application of wireless network technology and micro electro-mechanical systems, alongside the growing concern over energy supply constraints [1]. A piezoelectric vibration energy harvester is a device designed to capture mechanical vibrational energy from the surrounding environment, convert it into electrical energy, and either directly power electronic devices or store it in energy storage units [2].

Environmental vibration frequencies typically fall within the low-frequency range [3,4]. Classical cantilever beam structures typically only utilize the first-order resonance frequency, resulting in an uneven axial strain distribution with strain nodes [5]. These characteristics result in a lower energy capture density. To expand the bandwidth and enhance the power generation efficiency of the structure, various structural configurations have been proposed and analyzed [6,7]. Examples of such structural configurations include cross-sectional beam harvesters, tri-directional broadband harvesters, horizontally asymmetric U-shaped harvesters, fan-shaped vibration energy harvesters for leadless pacemakers, multi-directional harvesters for human motion, cross-shaped harvesters, and L-shaped beam piezoelectric energy harvesters [8,9].

The L-shaped energy harvester offers several advantages over the traditional cantilever beam piezoelectric energy harvester, including improved stability and rigidity, a simpler configuration, lower resonance frequencies, higher output responses, and easier integration into existing systems [10,11]. Furthermore, the L-shaped piezoelectric energy harvester cleverly utilizes the pivot structure of the L-shaped beam to achieve higher stability and load distribution, thereby enhancing the energy conversion efficiency [12,13,14]. This design is not only theoretically appealing but also demonstrates potential superiority in practical applications. Therefore, the L-shaped beam piezoelectric energy harvester is widely applied in fields such as renewable energy, structural health monitoring, and wireless sensor networks. The development of this technology offers new possibilities for the advancement of sustainable energy and intelligent sensing systems.

Erturk et al. [15] combined L-shaped beam structures with piezoelectric patches for piezoelectric energy harvesting. At the same time, an electro-mechanical coupling linear distributed parameter model was established to analyze the electro-mechanical coupling effects of the L-shaped beam structure, and its application in the landing gear of unmanned aerial vehicles was described. Haddow et al. [16] conducted experimental and theoretical analyses on the vibration responses of L-shaped beam structures at different excitation frequencies, focusing on the first two resonant frequencies. Nayfeh et al. [17] also conducted experimental studies on the vibration responses of L-shaped beams under harmonic excitation. The findings indicated that when the excitation frequency approached the lower resonant frequencies of the structure, periodic vibrations, quasi-periodic vibrations, and chaotic responses were observed. Xu et al. [18] developed a mechanical model for the axial strain distribution in the vertical beam of L-shaped beams, based on the Euler–Bernoulli beam theory and Hamilton’s principle. They conducted theoretical analyses and experimental studies, and also performed experimental analysis on the output power of the piezoelectric energy harvesting structure of L-shaped beams. Research indicates that the axial strain in the vertical beam of the L-shaped beam structure is more uniform, resulting in the higher utilization of the attached piezoelectric materials. Consequently, the output energy can reach twice that of traditional cantilever beams. Nie et al. [19] also conducted theoretical analysis and experimental validation on large deformation L-shaped energy harvesters. They analyzed the effects of external impedance and excitation amplitude on the displacement at the ends and energy output, as well as the geometric nonlinear effects.

Piezoelectric ceramics primarily serve as the material for the piezoelectric layer in the L-shaped piezoelectric vibration energy-harvesting device [20]. Given that piezoelectric ceramics are inorganic materials characterized by low electrical resistance and a poor quality factor, constructing an elastic layer is essential to enhance both strength and mechanical quality [21,22]. The elastic layer is vital for tuning the resonance frequency and boosting the energy harvesting efficiency of piezoelectric energy harvesters [23]. In the early stages of technological development, it is typically composed of isotropic metals or polymer materials [24]. Modern energy harvesters commonly utilize composite materials due to their favorable molding properties, cost-effectiveness, and design flexibility [25]. Commonly used composite materials include shape memory composites, symmetric angle–ply laminates, and fiber-reinforced composites [26,27].

In recent years, the composite material formed by 3D braided technology has emerged as a novel composite structure. Owing to their moldability, reduced production expenses, elevated specific strength and stiffness, and considerable design adaptability, they have seen extensive development and application in fields such as medical and sports equipment, marine structural engineering, aerospace engineering, and unmanned aerial vehicles.

Three-dimensional braided composite materials can optimize their vibration characteristics by adjusting their composition and structure. By precise design, the energy collector can be made more sensitive to mechanical vibration responses over a wider frequency range, thereby increasing the bandwidth of energy collection. Three-dimensional braided composite materials are usually lighter, which can reduce the mass of energy collectors, thereby improving their response speed and frequency range. This is particularly important for energy collectors that require frequent or rapid vibration, as it can expand their effective operating frequency range. Compared to conventional composite materials, the composite material formed by 3D braided technology employs a simpler molding process that allows for the rapid fabrication of materials with complex shapes and structures, thereby saving on production costs and time [28]. Three-dimensional braided technology allows for the integration of different fibers and matrix materials within the composite, enabling the fabrication of multifunctional composite materials [29]. Three-dimensional braided technology allows for the fabrication of materials with varying porosities and pore sizes, thereby meeting the requirements for different application scenarios [30]. The design space for the composite material formed by 3D braided technology is extensive, and it can be tailored to meet diverse engineering requirements by adjusting process parameters such as different fiber volume fractions [31,32]. Furthermore, the composite material formed by 3D braided technology enhances the interlaminar performance of laminated structures [33]. Due to their unique structural configuration, these materials also exhibit excellent energy absorption capabilities [34,35]. When subjected to impacts or vibrations, the interwoven fiber architecture can effectively disperse and absorb energy, providing enhanced protection for piezoelectric ceramics [36]. This results in a longer service life for the composite material formed by 3D braided technology structures [37,38]. Therefore, a novel L-shaped piezoelectric energy harvester based on the composite material formed by 3D braided technology is proposed in the article. The elastic layer of the device, which is composed of the composite material formed by 3D braided technology, is utilized to enhance the lateral mechanical properties of the structure. The design allows the braided angle of the composite elastic layer to be controlled, enabling the creation of harvesters with different resonant frequencies that are adaptable to various operational environments. In this paper, the first advantage of the L-shaped 3D BPCEH is that it can accept forces in two directions, so it can collect energy in different directions. Compared to traditional cantilever beams, it has a wider range of usage scenarios, such as wind power generation. Another advantage is that 3D braided composite materials have overall excellent elastic properties, such as good corrosion resistance and improved structural toughness.

The remaining parts of this article are as follows. In Section 2, an L-shaped 3D BPCEH model is established to predict the elastic properties of 3D braided composite under different braiding angles. In Section 3, the control equation of the L-shaped 3D BPCEH is derived, and the analytical solution for its voltage output is obtained. In Section 4, the influence of braiding angle and some key geometric parameters of the L-shaped three-dimensional braided composite material piezoelectric energy harvester on energy harvesting efficiency and voltage frequency response curve was analyzed. The conclusion can be found in Section 5.

## 2. The Model of L-Shaped 3D BPCEH

### 2.1. The Structural Model

**Figure 1:**
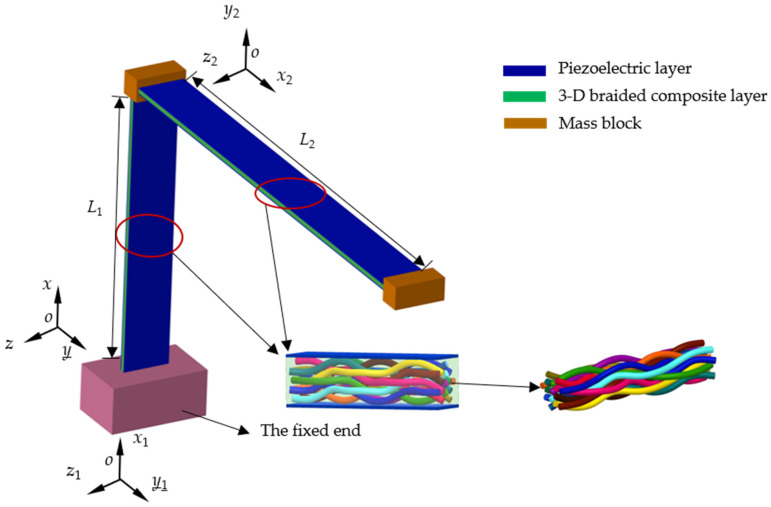
The sketch of the L-shaped 3D BPCEH.

This article introduces the design of the L-shaped 3D BPCEH, as depicted in Figure 1, with its dimensional parameters outlined in Table 1. Due to the significant length-to-thickness ratio of the piezoelectric cantilever beam, a mechanical model of the energy harvester is formulated based on the Euler–Bernoulli theory of linear beams.

The L-shaped 3D BPCEH includes cross beams, vertical beams, tuning mass blocks and fixed ends. The elastic layer of the L-shaped 3D BPCEH beam utilizes 3D braided composite, while the piezoelectric layer is composed of PZT-5H with material properties detailed in Table 2. The beams composed of composite material formed by 3D braided technology are based on solidification molding technology, combining prefabricated parts and matrix to obtain the composite material formed by 3D braided technology. The matrix of the composite material formed by 3D braided technology consists of epoxy resin, while the reinforcing fibers are composed of glass fiber-S.

### 2.2. Coupled Vibration Equations for the L-Shaped 3D BPCEH

#### 2.2.1. Motion Governing Equation for the L-Shaped 3D BPCEH

Regarding the electro-mechanical coupling effect in piezoelectric materials, the linear constitutive equation is as follows:

(1) $$D=eS+\varepsilon^{s}E$$

(2) $$T=c^{E}S-eE$$

where the symbol ***D*** presents the potential shift matrix, which quantifies the change in charge distribution. The matrix *ε^s^* represents the dielectric constant at constant strain, which reflects the degree of the polarization of the material under the action of an electric field. ***E*** refers to the electric field matrix, which describes the electric field strength inside the material. ***S*** represents the strain matrix inside the piezoelectric layer, which describes the deformation of the material after being stressed. ***T*** is the stress matrix, which represents the state of force endured within the material. ***c^E^*** is defined as the elastic stiffness constant matrix, which describes the material’s ability to resist deformation under a constant electric field. Finally, ***e*** represents the piezoelectric stress coefficient matrix under constant stress conditions, which reflects the characteristics of piezoelectric materials under the interaction of mechanical and electric fields.

Concerning the piezoelectric strain coefficient matrix ***d*** it is defined as follows:

(3) $$d=\left[\begin{array}{cccccc} 0 & 0 & 0 & d_{14} & d_{15} & 0\\ 0 & 0 & 0 & d_{24} & d_{25} & 0\\ d_{31} & d_{32} & d_{33} & 0 & 0 & d_{36} \end{array}\right]$$

**Figure 2:**
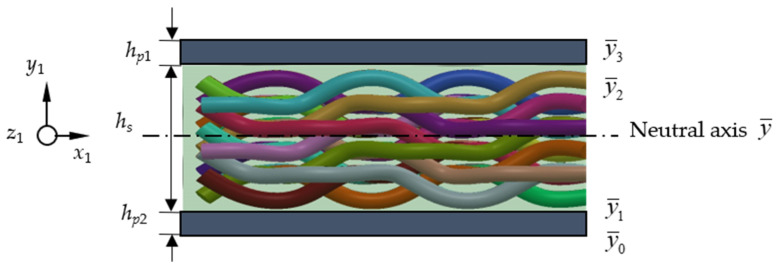
Neutral axis of 3D braided piezoelectric composite beam.

The lateral displacement of the L-shaped beam is assumed to be *w*(*x*), and its neutral axis is shown in Figure 2 (taking vertical beams as an example). The neutral axis of the beam is represented by $\bar{y}$. The neutral axis is the axis along which a beam experiences zero strain and stress during bending. In practical engineering, determining the position of the neutral axis is crucial for analyzing and designing the structural strength and stability of beams. By understanding the geometric and material properties of the cross-section, the position of the neutral axis can be calculated, thereby better understanding the deformation and stress distribution of the beam under stress conditions.

According to the equal moment of the upper and lower areas of the neutral axis, the distance from the neutral axis to the bottom boundary of the composite layer is calculated using the following method:

(4) $$\bar{y}=\frac{E_{p1}h_{p1}(h_{p1}+2h_{s1}+2h_{p2})+E_{s1}h_{s1}(h_{s1}+2h_{p1})+E_{p2}h_{p2}^{2}}{2(E_{p1}h_{p1}+E_{s1}h_{s1}+E_{p2}h_{p2})}$$

where *E_p_* and *E_s_* represent the elastic modulus of the piezoelectric layer and the composite layer; *h_p_* and *h_s_* represent the thickness of the piezoelectric sheet and the composite layer.

Therefore, in Figure 2, the interlayer position of the composite beam can be expressed as follows:

(5) $${\bar{y}}_{0}=-\bar{y},{\bar{y}}_{1}=h_{p2}-\bar{y},{\bar{y}}_{2}=h_{s1}+h_{p2}-\bar{y},{\bar{y}}_{3}=h_{p1}+h_{s1}+h_{p2}-\bar{y}$$

For the in-plane bending vibration of the L-shaped 3D BPCEH, the vibration displacements of the two beams can be expressed by *w_i_*(*x_i_*,*t*); then, the vibration control equation can be obtained as follows:

(6) $${\left(EI\right)}_{1}\frac{\partial^{4}w_{1}}{\partial x_{1}^{4}}+c\frac{\partial^{2}w_{1}}{\partial x_{1}^{2}}+m_{1}\frac{\partial^{2}w_{1}}{\partial t^{2}}+\Theta_{1}V_{1}(t)(\frac{\partial \delta }{\partial x_{1}}-\frac{\partial \delta }{\partial (x_{1}-L_{1})})=0$$

(7) $$\begin{array}{l} {\left(EI\right)}_{2}\frac{\partial^{4}w_{2}}{\partial x_{2}^{4}}+c\frac{\partial w_{2}}{\partial x_{2}}+m_{2}\frac{\partial^{2}w_{2}}{\partial t^{2}}+\Theta_{2}V_{2}(t)(\frac{\partial \delta }{\partial x_{2}}-\frac{\partial \delta }{\partial (x_{2}-L_{2})})\\ =-\left[m_{2}+m_{4}\delta (x_{2}-L_{2})-\frac{m_{4}}{2}L_{m2}\frac{\partial \delta }{\partial (x_{2}-L_{2})}\right]a_{x}(t) \end{array}.$$

In the formula, *x*_1_ and *x*_2_ are the length directions of the two beams, *L*_1_ and *L*_2_ are the lengths of the two beams, *V*_1_(*t*) and *V*_2_(*t*) represent the voltages across the two beams, *c*, *t*, *m*_2_ and *L_m_*_2_ represent, respectively, structural damping, time, free end tuning mass and free end tuning mass length, *δ* is the Dirac function, Θ_1_ is the electro-mechanical coupling term of the vertical beam, Θ_2_ is the electro-mechanical coupling term of the crossbeam, *m*_1_ and *m*_2_ denote the mass per unit length of the vertical and horizontal beams, *m*_3_ and *m*_4_ denote the masses of mass block A and mass block B, *J_m_*_1_ and *J_m_*_2_ denote the moments of inertia of mass blocks A and B, and *EI* represents bending stiffness.

In the L-shaped structure energy harvester, the potential shift equation of the piezoelectric layer can be described as follows:

(8) $$D_{3}=E_{p}d_{31}S_{1}^{P}+\varepsilon_{33}^{S}E_{3}$$

where $\varepsilon_{33}^{S}$ is the dielectric coefficient of the piezoelectric material and *E*_3_ is the voltage between the electrodes of the piezoelectric layer, which can be expressed as *E*_3_ = −*V*/*h_p_*. $S_{1}^{P}$ is the piezoelectric layer lengthwise strain term, which can be expressed as $S_{1}^{P}$ = −*y∂*^2^*w*/*∂x*^2^.

Based on the above, the piezoelectric layer potential shift equation is obtained as follows:

(9) $$D_{3}=E_{P}d_{31}(-y\frac{\partial^{2}w}{\partial x^{2}})+\varepsilon_{33}^{S}(-\frac{V}{h_{p}}).$$

Meanwhile, the boundary conditions satisfied by the bending vibration of the L-shaped 3D BPCEH are as follows:

when *x*_1_ = 0:

(10) $$w_{1}(0,t)=0,\frac{\partial w_{1}(0,t)}{\partial x_{1}}=0$$

(11) $$w_{2}(0,t)=0,\frac{\partial w_{2}(0,t)}{\partial x_{2}}=\frac{\partial w_{1}(0,t)}{\partial x_{1}}$$

when *x*_1_ = *L*_1_ or *x*_2_ = 0:

(12) $$\frac{\partial^{3}w_{1}}{\partial x_{1}^{3}}(L_{1},t)=\frac{m_{2}+m_{3}+m_{4}}{{\left(EI\right)}_{1}}\frac{\partial^{2}w_{1}(L_{1},t)}{\partial x_{1}^{2}}+\frac{(m_{2}+m_{3}+m_{4})h_{m1}}{2{\left(EI\right)}_{1}}\frac{\partial^{3}w_{1}(L_{1},t)}{\partial x_{1}\partial t^{2}}$$

(13) $$\frac{\partial^{2}w_{1}(L_{1},t)}{\partial x_{1}^{2}}=-\frac{J_{1}}{{\left(EI\right)}_{1}}\frac{\partial^{3}w_{1}(L_{1},t)}{\partial x_{1}\partial t^{2}}-\frac{m_{3}h_{m1}}{2{\left(EI\right)}_{1}}\frac{\partial^{3}w_{1}(L_{1},t)}{\partial x_{1}\partial t^{2}}+\frac{{\left(EI\right)}_{2}}{{\left(EI\right)}_{1}}\frac{\partial^{2}w_{2}(0,t)}{\partial x_{2}^{2}}$$

when *x*_2_ = *L*_2_:

(14) $$\frac{\partial^{3}w_{2}(L_{2},t)}{\partial x_{2}^{3}}=\frac{m_{4}}{{\left(EI\right)}_{2}}\frac{\partial^{2}w_{2}(L_{2},t)}{\partial x_{2}^{2}}+\frac{m_{4}L_{m2}}{2{\left(EI\right)}_{2}}\frac{\partial^{3}w_{2}(L_{2},t)}{\partial x_{2}\partial t^{2}}$$

(15) $$\frac{\partial^{2}w_{2}(L_{2},t)}{\partial x_{2}^{2}}=-\frac{J_{2}}{{\left(EI\right)}_{2}}\frac{\partial^{3}w_{2}(L_{2},t)}{\partial x_{2}\partial t^{2}}-\frac{m_{4}L_{m4}}{2{\left(EI\right)}_{2}}\frac{\partial^{2}w_{2}(L_{2},t)}{\partial x_{2}^{2}}$$

#### 2.2.2. Vibration Modal Analysis for the L-Shaped 3D BPCEH

It is determined that its vibration characteristics can be accurately described by modal analysis. This analysis highlights the system’s resonant frequencies and their corresponding mode shapes. In the process of model simplification, it is assumed that damping, external excitation, and the polarization effect of piezoelectric materials minimally impact the system’s response. These assumptions include setting the damping coefficient to *c* = 0, the excitation function to *a_x_*(*t*) = 0, and the piezoelectric potential differences *V*_1_(*t*) and *V*_2_(*t*) both to zero. Based on these parameters, the separation of the variables method is employed to express the lateral displacement of the beam, which is expressed as follows:

(16) $$w_{i}(x_{i},t)=\sum_{r=1}^{\infty }\phi_{ir}(x_{i})\eta_{r}(t)$$

where *ϕ_ir_*(*x_i_*) is the characteristic function of the vibration of the L-shaped 3D BPCEH at the *r*-order resonance frequency, *η_r_*(*t*) is the modal coordinate of the vibration of the L-shaped 3D BPCEH at the *r*-order resonance frequency, and *i* represents the number of the beam and takes the value 1 or 2.

By substituting Equation (16) into Equations (6) and (7) and the boundary conditions, respectively, the governing equations can be obtained as follows:

(17) $$\phi_{1}^{4}-\frac{m_{1}}{{\left(EI\right)}_{1}}\omega^{2}\phi_{1}=0$$

(18) $$\phi_{2}^{4}-\frac{m_{2}}{{\left(EI\right)}_{2}}\omega^{2}\phi_{2}=0$$

According to the governing Equation (17), Equation (18) and the boundary conditions, the special solution of the governing equation can be obtained as follows:

(19) $$\phi_{1}(x_{1})=C_{1}(\sin ax_{1}-\sinh ax_{1})+C_{2}(\cos ax_{1}-\cosh ax_{1})$$

(20) $$\phi_{2}(x_{2})=D_{1}\sin\lambda x_{2}+D_{2}(\cos\lambda x_{2}-\cosh\lambda x_{2})+D_{3}\sin\lambda x_{2}$$

where, *C*_1_, *C*_2_, *D*_1_, *D*_2_ and *D*_3_ represent the modal coefficients of the characteristic function, *a* and *λ* represent the correlation coefficients of frequency *ω*. It can be seen that the appearance of negative signs in the special solution of the control equation is determined by the deformation direction and the boundary conditions of the beam under stress. These factors can affect the form of the deflection equation, leading to negative signs in specific situations.

#### 2.2.3. Steady-State Response Analysis for the L-Shaped 3D BPCEH

The L-beam structural vibration energy harvester is subjected to in-plane bending vibration under a simple harmonic excitation *a_x_*(*t*) = *asin*(*ωt*); then, its governing equation can be written as follows:

(21) $$\frac{\partial^{2}\eta_{r}}{\partial t^{2}}+2\xi_{r}\omega_{r}\frac{d\eta_{r}}{dt}+\omega_{r}^{2}\eta_{r}+\sum_{i=1}^{2}\chi_{ri}V_{i}=f_{r}(t)$$

where *ξ_r_* represents the damping ratio of the *r*-th mode, *f_r_*(*t*) represents the force load function in the modal coordinates, and *χ_ri_* corresponds to the electromechanical coupling term in the modal coordinates. The specific forms of *f_r_*(*t*) and *χ_ri_* are as follows:

(22) $$f_{r}(t)=-\int_{0}^{L_{2}}m_{2}\phi_{2rm}a_{x}dx_{2}-m_{4}a_{x}\phi_{2rm}(L_{2})-\frac{m_{4}}{2}L_{m2}a_{x}\frac{d\phi_{2rm}}{dL_{2}}$$

(23) $$\chi_{r}=\Theta_{i}\frac{d\varphi_{ir}(x)}{dx}{|}_{x=L_{i}}.$$

Based on Gauss’s law and Kirchhoff’s law, the current flowing through the piezoelectric layer is defined as follows:

(24) $$I=\frac{d}{dt}\int_{A}D\cdot ndA=\frac{V}{R}.$$

Combining Equations (9) and (24), the electric field equations of the two beams can be obtained as follows:

(25) $$-(E_{p1}d_{31}^{p1}h_{pc1}+E_{p2}d_{31}^{p2}h_{pc2})b\int_{0}^{L_{1}}\frac{\partial^{3}w_{1}(L_{1},t)}{\partial x_{1}\partial t^{2}}dx_{1}-bL_{1}(\frac{\varepsilon_{33}^{Sp1}}{h_{p1}}+\frac{\varepsilon_{33}^{Sp2}}{h_{p2}})\frac{dV_{1}}{dt}=\frac{V_{1}(t)}{R_{1}}$$

(26) $$-(E_{p3}d_{31}^{p3}h_{pc3}+E_{p4}d_{31}^{p4}h_{pc4})b\int_{0}^{L_{2}}\frac{\partial^{3}w_{2}(L_{2},t)}{\partial x_{2}\partial t^{2}}dx_{2}-bL_{2}(\frac{\varepsilon_{33}^{Sp3}}{h_{p3}}+\frac{\varepsilon_{33}^{Sp4}}{h_{p4}})\frac{dV_{2}}{dt}=\frac{V_{2}(t)}{R_{2}}$$

where *b* represents the width of the structure.

The following equations are available after collation:

(27) $$C_{p1}\frac{dV_{1}}{dt}+\frac{V_{1}(t)}{R_{1}}=\sum_{r=1}^{\infty }K_{1r}{\dot{\eta }}_{r}$$

(28) $$C_{p2}\frac{dV_{2}}{dt}+\frac{V_{2}(t)}{R_{2}}=\sum_{r=1}^{\infty }K_{2r}{\dot{\eta }}_{r}$$

where *C_p_*_1_ and *C_p_*_2_ represent the capacitances of the piezoelectric layers for the two beams, while *K*_1*r*_ and *K*_2*r*_ are the *r*-order mode coupling terms of the two beams. The specific expression is as follows:

(29) $$C_{p1}=bL_{1}(\frac{\varepsilon_{33}^{S_{p1}}}{h_{p1}}+\frac{\varepsilon_{33}^{S_{p2}}}{h_{p2}}),C_{p2}=bL_{2}(\frac{\varepsilon_{33}^{S_{p3}}}{h_{p3}}+\frac{\varepsilon_{33}^{S_{p4}}}{h_{p4}})$$

(30) $$K_{1r}=-(E_{p1}d_{31}^{p1}h_{pc1}+E_{p2}d_{31}^{p2}h_{pc2})b{\phi^{\prime }}_{1rm}$$

(31) $$K_{2r}=-(E_{p3}d_{31}^{p3}h_{pc3}+E_{p4}d_{31}^{p4}h_{pc4})b{\phi^{\prime }}_{2rm}.$$

Under simple harmonic excitation, the expressions of modal coordinates, voltage and acceleration are as follows:

(32) $$\eta_{r}(t)=H_{r}e^{j\omega t},V_{i}(t)=U_{i}e^{j\omega t},a_{x}(t)=a_{bx}e^{j\omega t}$$

substituting it into Equation (21), we can obtain the following:

(33) $$H_{r}=\frac{f_{r0}-\sum_{i=1}^{2}\chi_{ri}U_{ir}}{\omega_{r}^{2}-\omega^{2}+2\xi_{r}\omega_{r}\omega j}$$

where *H_r_* is the complex number of the modal coordinates in the *r*-order mode, *U_ir_* is the complex number of the output voltages of the two beams, and *f_r_*_0_ represents the complex number associated with the modal load function.

Combining Equations (23), (27), (28) and Equation (33), we can obtain the voltage of the two beams under *r*-order vibration:

(34) $$U_{1r}=\frac{Z_{3}Z_{5}-Z_{2}Z_{6}}{Z_{1}Z_{5}-Z_{2}Z_{4}}$$

(35) $$U_{2r}=\frac{Z_{1}Z_{6}-Z_{3}Z_{4}}{Z_{1}Z_{5}-Z_{2}Z_{4}}.$$

The specific forms of *Z*_1_~*Z*_6_ in the formula are as follows:

(36) $$Z_{1}=C_{p1}\omega j+\frac{1}{R_{1}}+\sum_{r=1}^{\infty }\frac{\omega j}{\omega_{r}^{2}-\omega^{2}+2\xi_{r}\omega_{r}\omega j}K_{1r}\chi_{r1}$$

(37) $$Z_{2}=\sum_{r=1}^{\infty }\frac{\omega j}{\omega_{r}^{2}-\omega^{2}+2\xi_{r}\omega_{r}\omega j}K_{1r}\chi_{r2}$$

(38) $$Z_{3}=\sum_{r=1}^{\infty }\frac{\omega j}{\omega_{r}^{2}-\omega^{2}+2\xi_{r}\omega_{r}\omega j}K_{1r}f_{r0}$$

(39) $$Z_{4}=\sum_{r=1}^{\infty }\frac{\omega j}{\omega_{r}^{2}-\omega^{2}+2\xi_{r}\omega_{r}\omega j}K_{2r}\chi_{r1}$$

(40) $$Z_{5}=C_{p2}\omega j+\frac{1}{R_{2}}+\sum_{r=1}^{\infty }\frac{\omega j}{\omega_{r}^{2}-\omega^{2}+2\xi_{r}\omega_{r}\omega j}K_{2r}\chi_{r2}$$

(41) $$Z_{6}=\sum_{r=1}^{\infty }\frac{\omega j}{\omega_{r}^{2}-\omega^{2}+2\xi_{r}\omega_{r}\omega j}K_{2r}f_{r0}$$

## 3. The Mechanical Characteristics of the 3D BPCEH Elastic Layer

**Figure 3:**
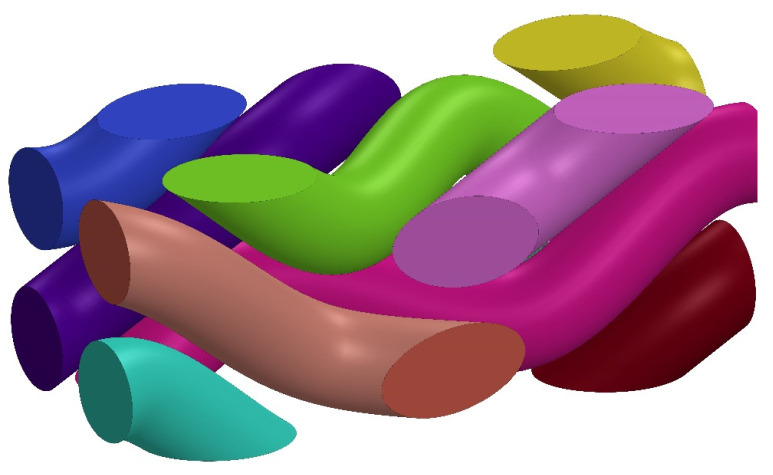
The fiber model of the composite formed by 3D braided technology.

**Figure 4:**
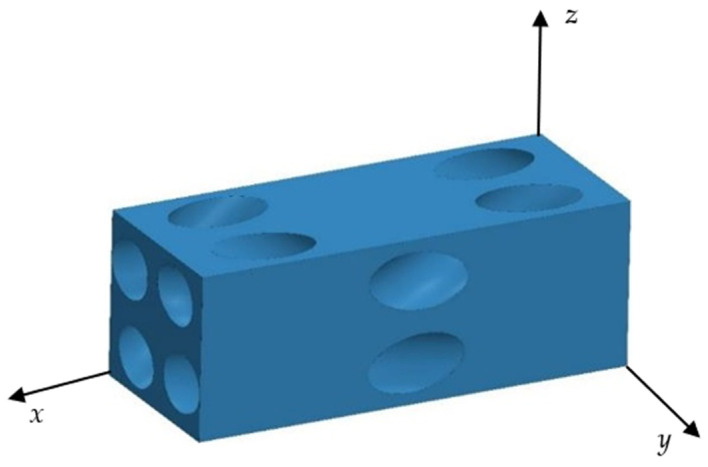
The matrix model of the composite formed by 3D braided technology.

**Figure 5:**
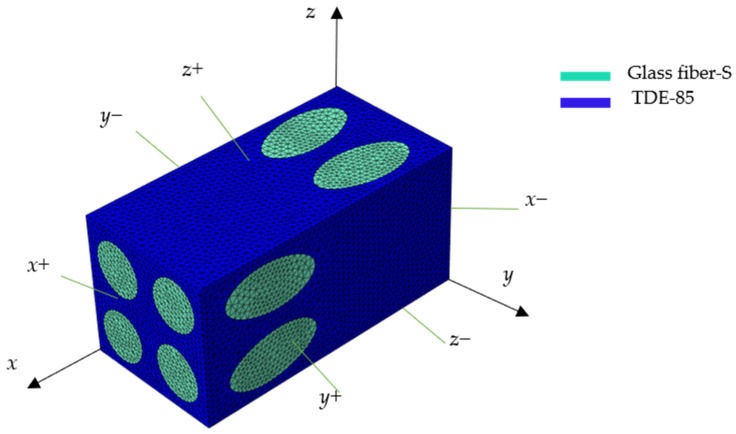
The FEM model of the RVE.

Utilizing the specialized four-step braided process, the structure can be designed in a variety of configurations. In this study, the four-step braided technique is adopted, and SolidWorks 2019 is used to construct the geometric model of the prefabricated parts. By adjusting the braiding angle of the internal fibers, based on the composite material formed by 3D braiding with different braided angles (20°, 25°, 30°, 35° and 40°), their material properties are predicted and comparatively analyzed. In this study, a braided flower node is selected as an RVE of the macro prefabricated part in order to carry out the performance analysis. For example, in the case of the composite materials based on 3D braided technology with a braided angle of 35°, its unit cell fibers are shown in Figure 3, its unit cell matrix is shown in Figure 4, the material parameters of the glass fibers and the epoxy resin are shown in Table 3, and the finite element model of its RVE is depicted in Figure 5. In order to ensure the continuity of the model, periodic boundary conditions are applied on the RVE during the finite element simulation, which are detailed in Table 4. The six faces of the RVE are identified with the symbols (*x+*, *x*−, *y+*, *y*−, *z+*, *z*−).

During the finite element analysis process, the matrix material is discretized using C3D10E cells with the total number of meshes up to 204,356, while the fiber bundles are delineated using C3D8R cells with the mesh number of 36,598. The material parameters of composite material formed by 3D braiding technology are further calculated and analyzed for different braided angles, namely 20°, 25°, 30°, 35° and 40°, and the relevant data are summarized in Table 5. In addition, this article provides insights into how the variation of the braided angle affects the mechanical properties of the composites, specifically investigating key mechanical parameters including elastic modulus, shear modulus, and Poisson’s ratio.

By comparing the material parameters under different braided angles, the article aims to reveal the effect of the braided angle on the mechanical properties of the composite material formed by 3D braiding technology, which provides theoretical basis and reference for the design and application of composites. In the analysis of the data in Table 5, we observe a significant trend, namely as the braided angle is raised from 20° to 40°, a significant decrease in the probed axial modulus of elasticity *E*_11_ is observed, from 28.49 GPa to 18.59 GPa. Meanwhile, the transverse modulus of elasticity *E*_33_ showed the opposite trend, increasing from 11.21 GPa to 14.87 GPa. The reason is that the increase in the braided angle leads to an increase in the volume fraction of transverse fibers and a relative decrease in the volume fraction of axial fibers. Specifically, as the braided angle expands, the coverage and cross-density of fiber bundles in the transverse direction increase, making the fibers in this direction become denser and stronger, thereby increasing the rigidity of the composite material in this direction. This change in the geometric distribution of fibers directly affects the load transfer path and elastic response of the material. Since fibers are the main load-bearing elements in composites, fiber thickening in the transverse direction results in an increase in the elastic modulus in this direction. On the contrary, due to the relative sparseness of the fibers in the axial direction, its load-bearing capacity decreases, resulting in a decrease in the axial elastic modulus.

## 4. Output Response Analysis of the L-Shaped 3D BPCEH

### 4.1. Effect of the Mass of Mass Block B on the Resonant Frequency of L-Shaped 3D BPCEH

In the field of the composite material formed by 3D braiding technology, different braided angles will lead to differences in their mechanical properties. The main research content of this section is to comparatively analyze the output performance of L-shaped 3D BPCEH with braided angles of 20°, 25°, 30°, 35° and 40° under the same load and excitation amplitude. The first three resonant frequencies of the L-shaped 3D BPCEH are calculated and presented in Table 6. As the braided angle continues to increase, the first three-order resonant frequencies show a decreasing trend. As the braided angle increases, the axial properties of the composite material formed by 3D braiding technology decrease, while the transverse properties improve. This change leads to corresponding adjustments in the axial elastic modulus and transverse modulus of the material, which in turn affects the resonant frequency of the device.

**Figure 6:**
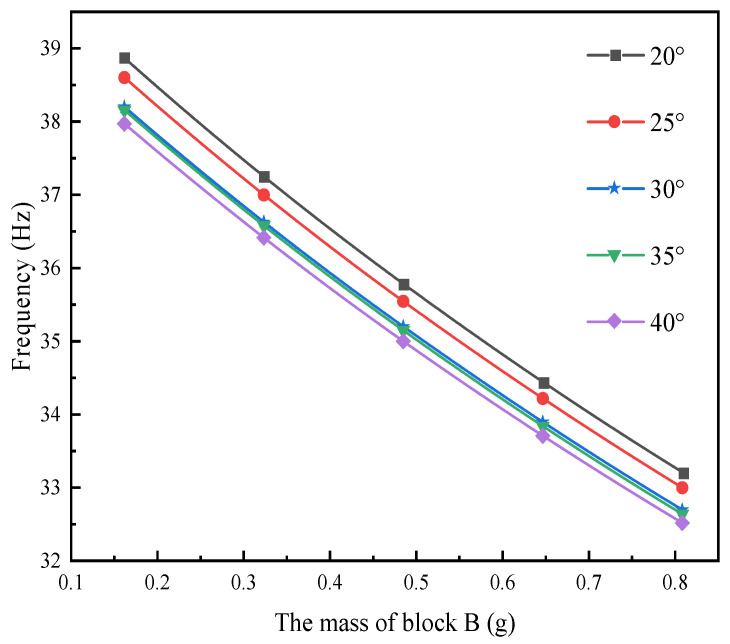
The variation in the first-order resonant frequency with changes in mass block B under different braided angles.

**Figure 7:**
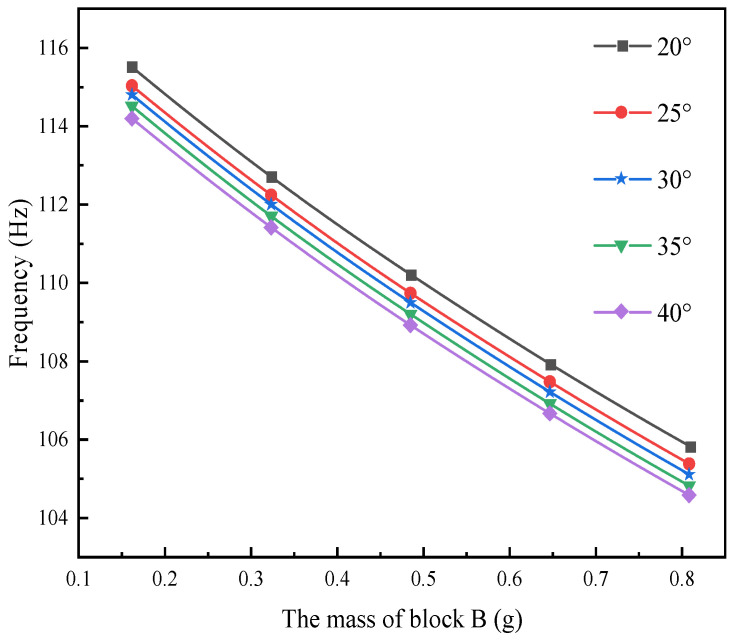
The variation in the second-order resonant frequency with changes in mass block B under different braided angles.

**Figure 8:**
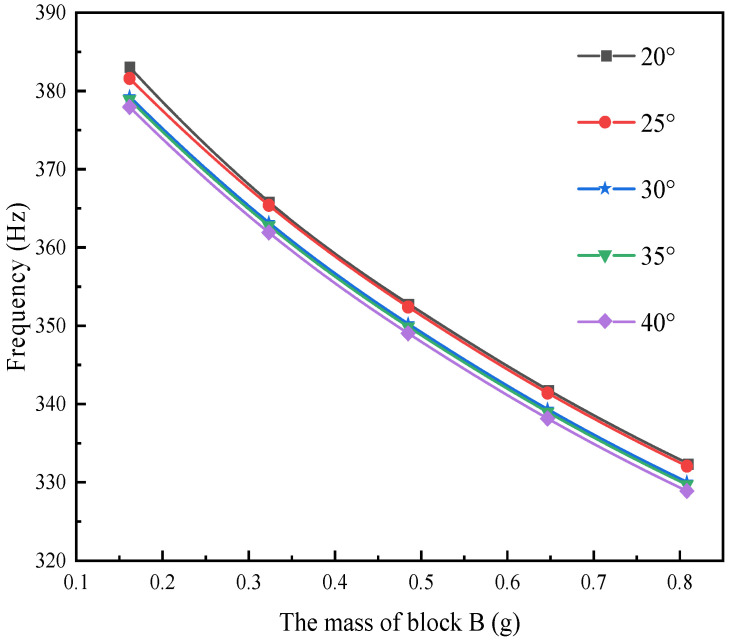
The variation in the third-order resonant frequency with changes in mass block B under different braided angles.

The effect of varying the mass of block B on the first three resonant frequencies of the L-shaped 3D BPCEH, which features different braiding angles, is analyzed by adjusting the thickness of block B while keeping the mass of block A constant. The results are displayed in Figure 6, Figure 7 and Figure 8.

The figure demonstrates a clear phenomenon; the first three orders of the resonant frequency of the L-shaped 3D BPCEH gradually decrease as the mass of mass block B keeps increasing. The data indicate that by increasing the mass of mass block B, the resonant frequency can be effectively reduced, thereby facilitating the ability of the L-shaped 3D BPCEH to capture environmental energy more efficiently.

### 4.2. Voltage-Frequency Domain Response Analysis of L-Shaped 3D BPCEH under Different Excitation Directions

**Figure 9:**
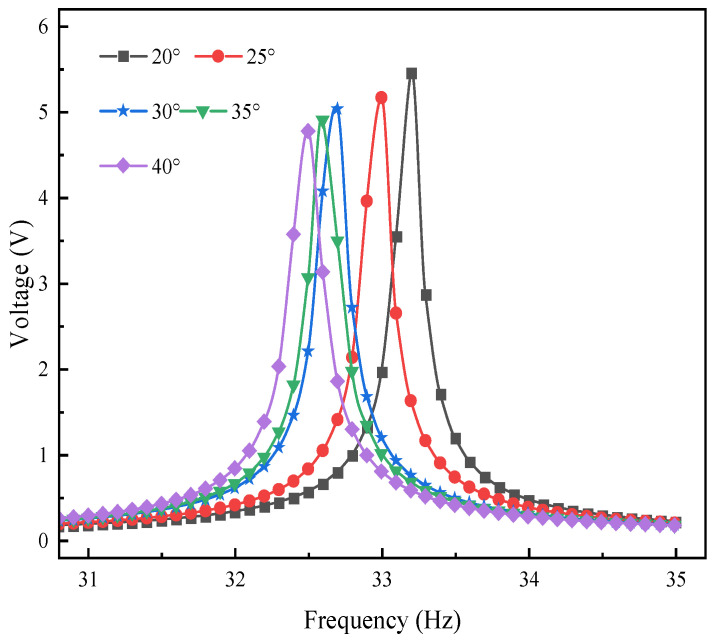
The variation of voltage with excitation frequency under *x*-direction.

**Figure 10:**
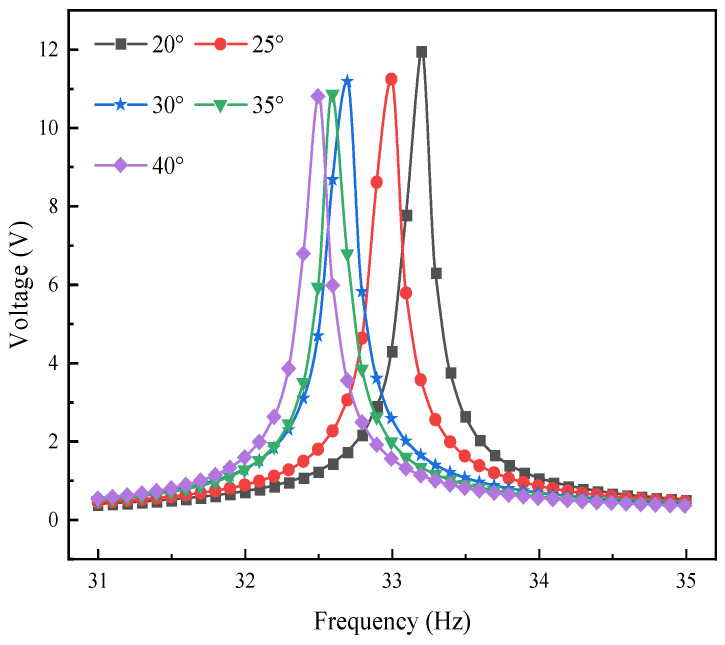
The variation of voltage with excitation frequency under *y*-direction.

In the study of the performance of L-shaped 3D BPCEH, the influence of different braiding angles on the voltage–frequency domain response at the first-order resonant frequency is primarily examined. In the simulation setup, the input resistance of the device is fixed at 1 kΩ, and the excitation acceleration is 1 g. Through simulation, changes in the voltage–frequency domain response are analyzed when excitation is applied in the *x*-direction and *y*-direction. The specific results are displayed in Figure 9 and Figure 10. The study found that as the braided angle gradually decreases, the peak voltage of the device shows a decreasing trend regardless of the excitation in the *x*-direction or the *y*-direction. This phenomenon reveals the braided angle as a key parameter in the vibration energy harvester of L-shaped 3D braided piezoelectric composites, which has a significant impact on the voltage output and resonant frequency. Specifically, different braided angles will cause changes in material properties, thereby changing the first-order resonant frequency of the system. As the braided angle is adjusted, the resonant frequency shows a downward trend, which further confirms that the braided angle is an effective means to adjust the voltage response and system resonance characteristics. When comparing the effects of excitation applied in the *y*-direction and *x*-direction, it is observed that the output voltage generated by the device under excitation in the *y*-direction is significantly higher than that in the *x*-direction. This finding indicates that the output voltage of the device is influenced not only by the braided angle but is also closely related to the direction of excitation.

### 4.3. Effect of Load Resistance on the Output Response of L-Shaped 3D BPCEH

The purpose of this section of the research is to deeply explore the impact of different load resistances on the output response of the L-shaped 3D BPCEH and its changing trend under the condition of a 35° braided angle. Through simulation analysis, the response curves of output power and voltage changing with frequency under a series of load resistance values are drawn. These curves reveal the optimal performance of the L-shaped 3D BPCEH under different conditions, and through these analyses. It is anticipated that the design of the L-shaped 3D BPCEH will be optimized to achieve maximum energy conversion efficiency in various practical application environments.

**Figure 11:**
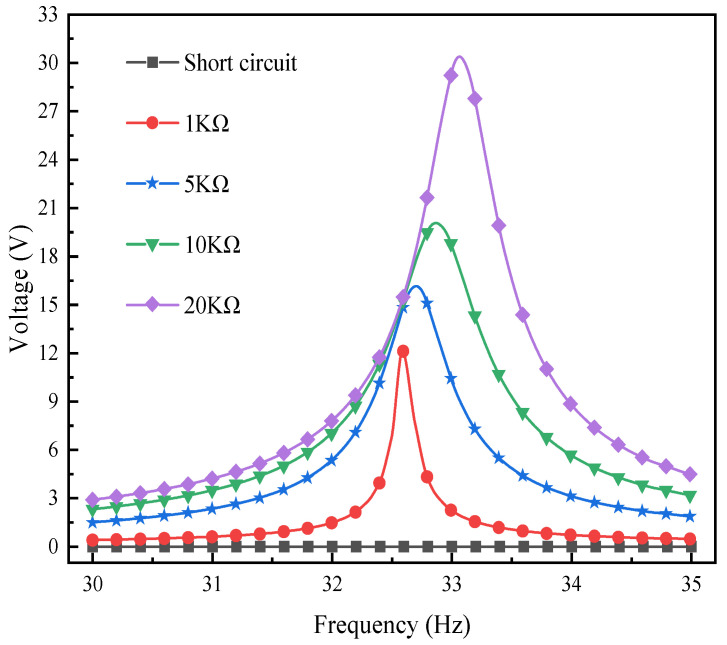
The changes in voltage response under different load resistances near the first-order resonant frequency.

**Figure 12:**
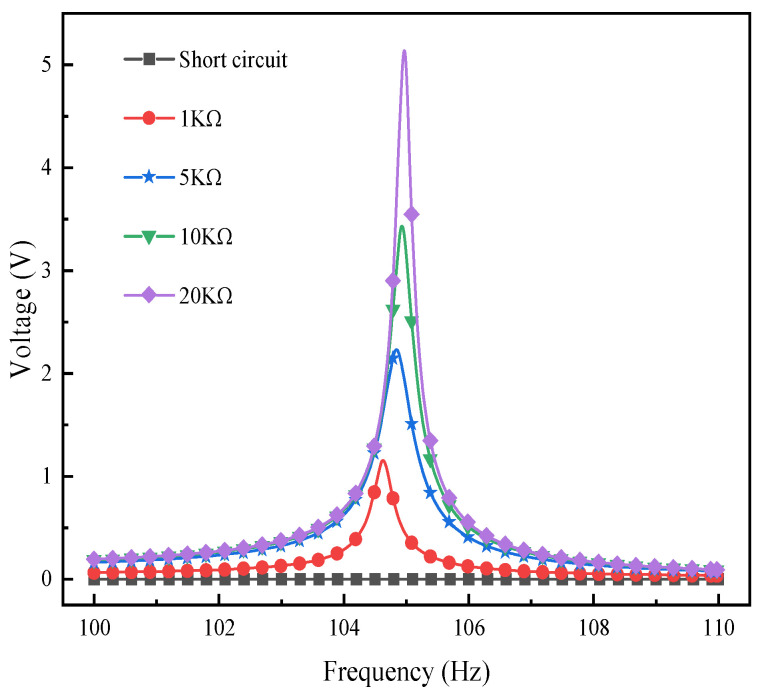
The changes in voltage response under different load resistances near the second-order resonant frequency.

Figure 11 depicts the voltage output response of the L-shaped 3D BPCEH with the braided angle set at 35° for different load resistance in the first-order frequency domain, while Figure 12 reflects the voltage output response in the second-order frequency domain. Around the first-order resonant frequency, the observation of the data reveals that the voltage response of the L-shaped 3D BPCEH with 35° braided angle shows an upward trend as the load resistance increases, while the resonant frequency is shifted with the load resistance. When the input resistance is 1 kΩ, the peak voltage is 12.066 V and the resonance frequency is 32.6 Hz. When the input resistance is 5 kΩ, the peak voltage is 16.095 V and the resonance frequency is 32.7 Hz. When the input resistance is 10 kΩ, the peak voltage is 19.959 V and the resonance frequency is 32.9 Hz. When the input resistance is 20 kΩ, the peak voltage is 30.202 V and the resonant frequency is 33.1 Hz. The larger the load resistance, the more pronounced the shift in resonant frequency. For all values of load resistance, the peak voltage output is close to the resonant frequency of the structure. These data demonstrate how load resistance affects energy harvesting efficiency and the resonant characteristics of the system. This finding suggests that the load resistance significantly affects both the voltage output response and the resonant frequency of the L-shaped 3D BPCEH.

### 4.4. Effect of Excitation Acceleration on the Output Response of the L-Shaped 3D BPCEH

**Figure 13:**
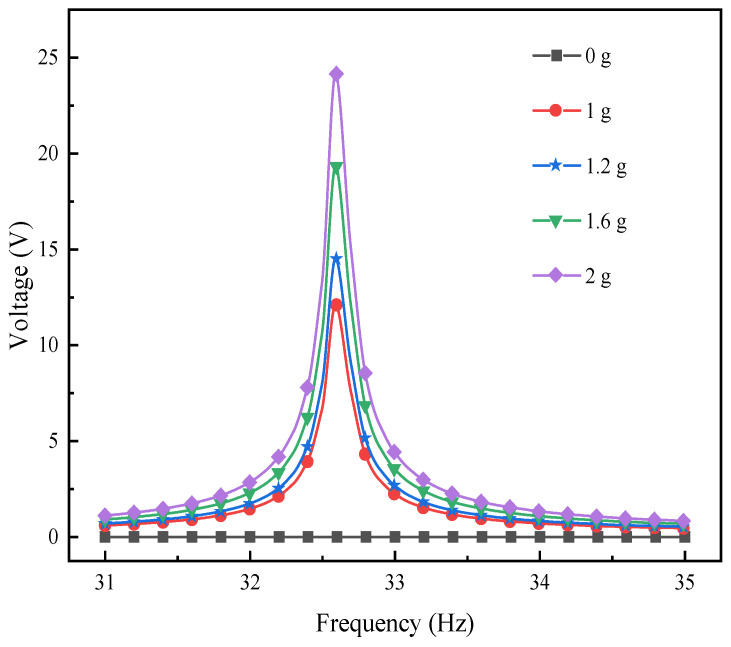
The changes in voltage response under different external excitation acceleration near the first-order resonant frequency.

**Figure 14:**
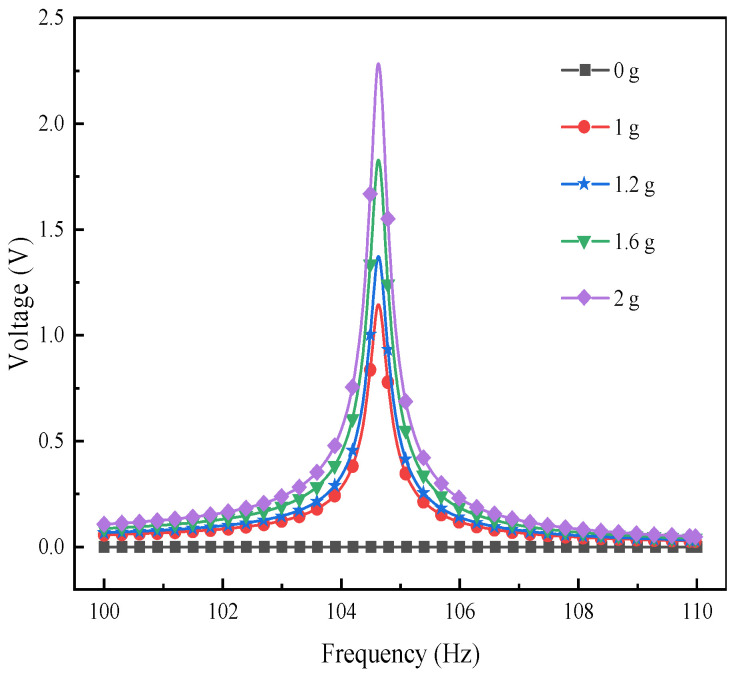
The changes in voltage response under different external excitation acceleration near the second-order resonant frequency.

Figure 13 illustrates the impact of varying excitation accelerations on the output voltage of the L-shaped 3D BPCEH within 31~34.5 Hz in the first-order mode analyzed at a braided angle of 35°, and Figure 14 shows the impact of different excitation acceleration on the output voltage of the L-shaped 3D BPCEH within 100~110 Hz in the second-order mode analyzed at a braided angle of 35°. From Figure 13, it can be found that as the excitation acceleration increases from 1 g to 2 g, the maximum output voltage increases from 12 V to 24.6 V, and consistently peaks near the resonant frequency of 32.6 Hz. From Figure 14, it can be found that as the excitation acceleration increases from 1 g to 2 g, the maximum output voltage increases from 1.1 V to 2.2 V, and invariably occurs close to the resonance frequency of 104.64 Hz, so it can be seen that the excitation acceleration only affects the output voltage of the L-shaped 3D BPCEH and cannot affect its resonance frequency.

**Figure 15:**
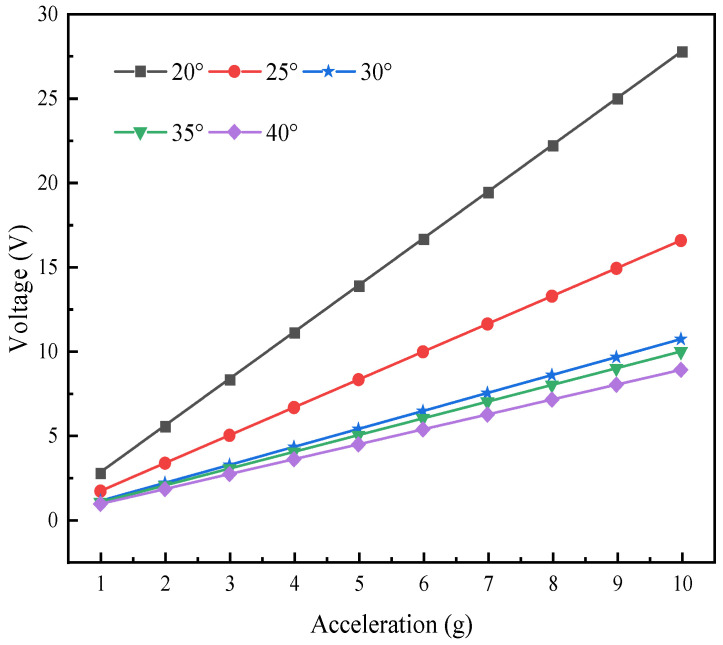
The voltage changes with acceleration.

The voltage output response of the L-shaped 3D BPCEH under different excitation accelerations and braided angles is analyzed, with the simulation results displayed in Figure 15. It can be found that for the same excitation acceleration, the output voltage decreases as the braided angle keeps increasing; for the same braided angle, the output voltage increases as the excitation acceleration keeps increasing.

**Figure 16:**
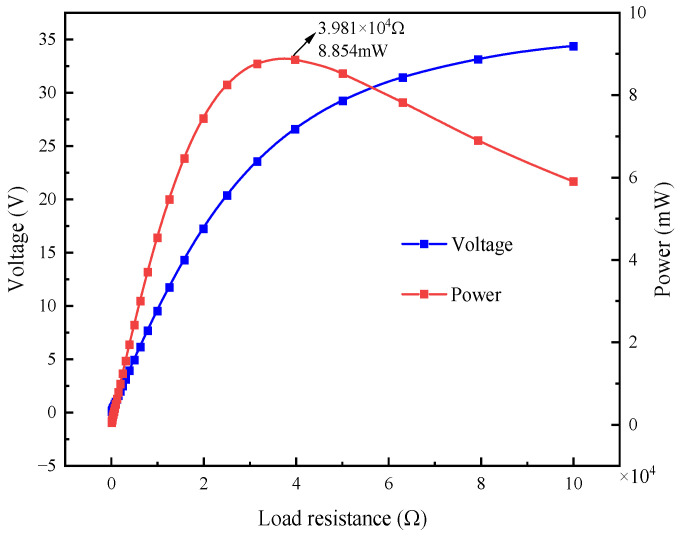
The voltage and power changes with the load resistance.

As shown in Figure 16, when the L-shaped 3D BPCEH with the braided angle set to 35° receives different loads, the variation rules of its output voltage and power are clearly depicted. For the L-shaped 3D BPCEH with the braided angle of 35°, the output voltage exhibits an overall upward trend as the external resistance increases, in which the growth rate first rises rapidly and then gradually stabilizes. Correspondingly, the output power first rises to the peak value as the external load increases, and then begins to decrease. In particular, when the external resistance is 39.81 kΩ, the output power of the L-shaped 3D BPCEH reaches the peak value, which is 8.854 mW. It can be inferred that the equivalent internal resistance of an L-shaped 3D BPCEH with the braided angle of 35° is approximately 39.81 kΩ.

### 4.5. Effect of the Mass of Mass Block B on the Output Response of the L-Shaped 3D BPCEH

**Figure 17:**
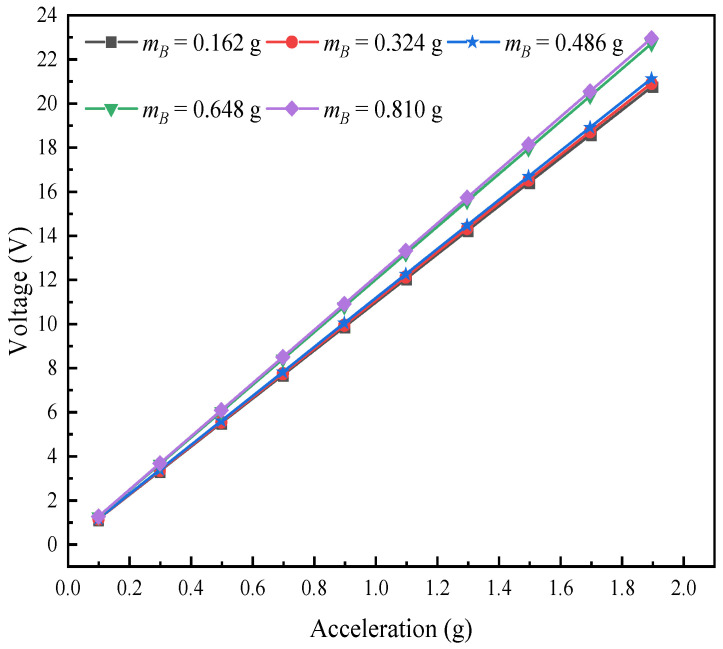
The variation of voltage with mass block B under different accelerations.

This research chapter analyzes the voltage response and changing rules of the L-shaped 3D BPCEH under the condition of changing the mass of mass B at the fixed braided angle, taking the braided angle of 35° as a research case. In the setup, the input resistance is fixed at 1 kΩ, while the excitation frequency corresponds to the first-order resonant frequency of the 35° braided angle L-shaped 3D BPCEH, which is 32.6 Hz. Figure 17 demonstrates the trend of the effect of different excitation accelerations on the output voltage of the L-shaped 3D BPCEH for a variety of mass block B masses. The data in the figure reveal a clear trend; namely as the mass of mass block B gradually increases, the output voltage of the L-shaped 3D BPCEH increases accordingly. This is probably because the increase in the mass block makes the vibration amplitude of the device increase at the excitation frequency, which may lead to more stress acting on the piezoelectric material, thus generating a higher voltage. And when the excitation acceleration is gradually increased, the output voltage shows a proportional increase, following a linear relationship as a primary function.

**Figure 18:**
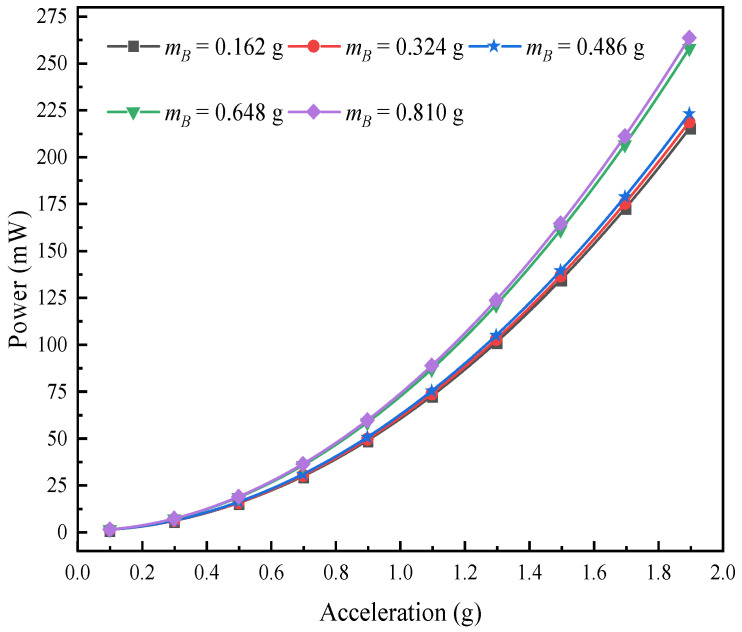
The variation of power with mass block B under different accelerations.

Similarly, the effect of different excitations on the output power of the L-shaped 3D BPCEH is analyzed for different masses of mass block B. The results of the analysis are shown in Figure 18. It can be observed that as the mass of mass block B keeps on increasing, the output power also keeps on increasing, which is in good agreement with the voltage variation. As the excitation acceleration keeps increasing, the output power tends to increase in a quadratic relationship.

**Figure 19:**
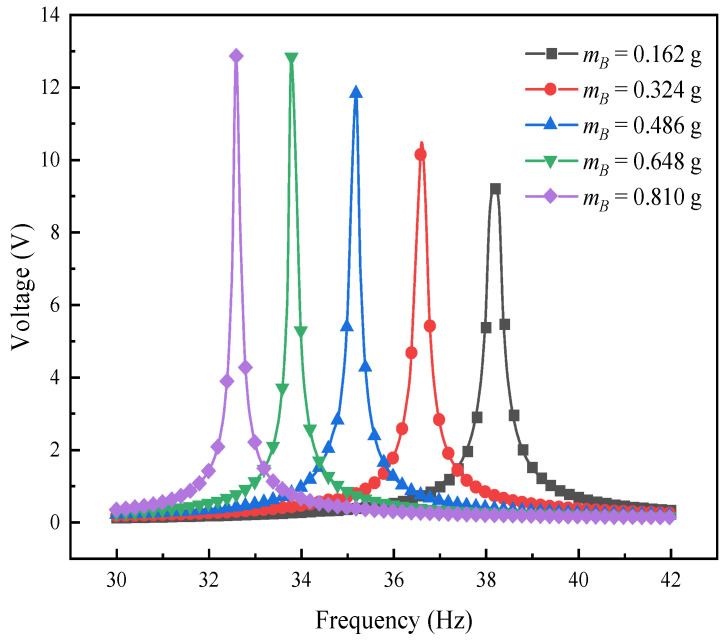
The variation of voltage with mass block B near the first-order resonant frequency.

**Figure 20:**
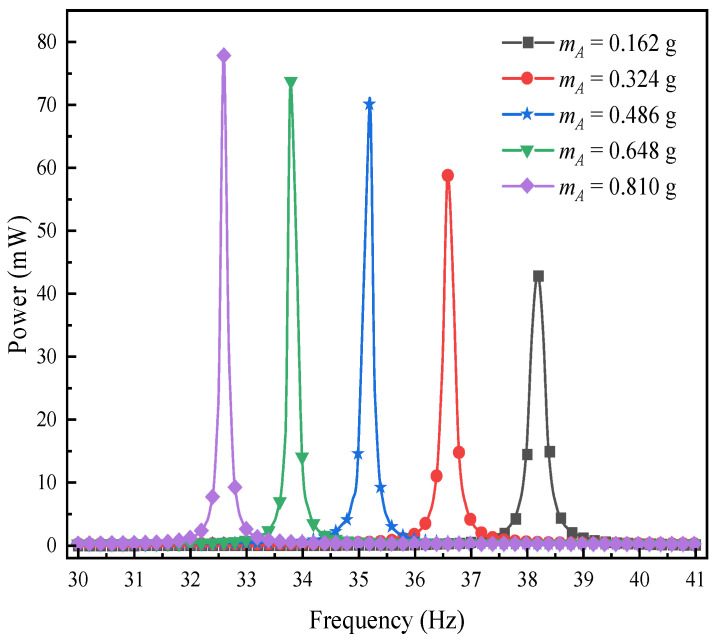
The variation of power with mass block B near the first-order resonant frequency.

To gain a deeper understanding of how the mass of mass block B affects the performance of the L-shaped 3D BPCEH, this study plotted the response curves of the output voltage and output power by sweeping the frequency analysis near the first-order resonant frequency (30~42 Hz). The relevant results are presented in Figure 19 and Figure 20, respectively. Observing the obtained data curves, it can be clearly seen that the first-order resonant frequency of the L-shaped 3D BPCEH decreases gradually as the mass of mass block B gradually increases. At the same time, both the peak voltage and power show a gradual increase in the trend of change. The reason is that changing the thickness of mass block B results in a shift in the overall resonant frequency of the entire device, which in turn affects the resonance frequency, output voltage and output power. Therefore, in practice, we can adjust the resonant frequency, voltage output and power output of the whole device by changing the mass of mass block B.

### 4.6. Effect of Beam Length on the Output Response of the L-Shaped 3D BPCEH

This subsection explores the output response and response change rule of L-shaped 3D BPCEH under the condition of different beam lengths with fixed braided angle. The braided angle set in this article is 35°, and analyzes the effect of beam length on the first three orders of the resonant frequency of L-shaped 3D BPCEH, and the results are shown in Table 7. As the beam length increases from 30 mm to 100 mm, the first-order resonance frequency of the L-shaped 3D BPCEH decreases from 88.752 Hz to 32.648 Hz, the second-order resonance frequency from 303.81 Hz to 104.64 Hz, and the third-order resonance frequency from 843.88 Hz to 328.96 Hz, all of which are in the trend of continuous decrease.

**Figure 21:**
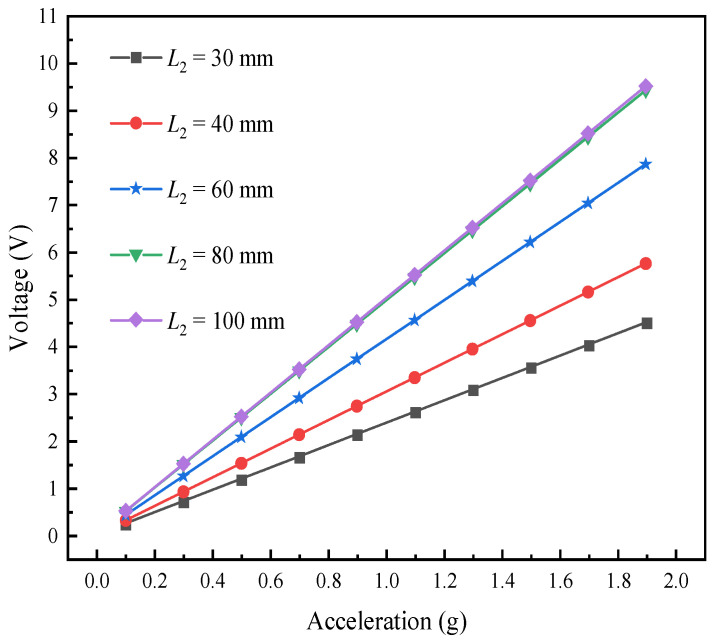
The variation of voltage with beam length under different accelerations.

The performance of the L-shaped 3D BPCEH is analyzed at the fixed external resistance of 1 kΩ, a specific braided angle of 35°, and an excitation frequency that is set to the first-order resonant frequency of the device corresponding to different beam lengths. Figure 21 demonstrates how different excitation accelerations affect the output voltage of the L-shaped 3D BPCEH under varying beam lengths. These data provide a reference for analyzing the effect of beam length on the efficiency of energy harvesting and help guide how to adjust the device parameters to optimize performance.

**Figure 22:**
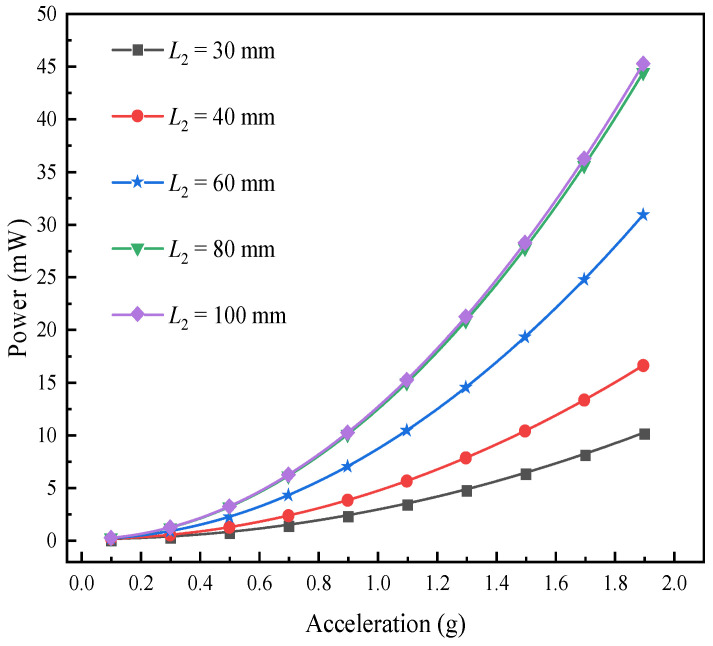
The variation of power with beam length under different accelerations.

Figure 22 shows the effect of different excitation accelerations on the power output response of the L-shaped 3D BPCEH for different beam lengths. It can be found that the output voltage and power of the L-shaped 3D BPCEH are in the trend of increasing as the beam length keeps increasing. Therefore, by appropriately increasing the length of the beam during the design and application process, we can not only improve the collection efficiency of the energy harvester, but also refine the output response of the device through the adjustment of such structural parameters. A longer beam helps to capture more energy, thus increasing the output voltage and power; on the other hand, it may lead to a decrease in the resonant frequency, which needs to be matched to the operating frequency of the energy harvester to ensure the most efficient energy conversion.

## 5. Conclusions

In this article, an innovative L-shaped 3D BPCEH with superior mechanical performance is designed, and the mechanical characteristics of the L-shaped 3D BPCEH under various braided angles are examined. With an increase in braided angles, there is a decrease in axial mechanical characteristics and an increase in transversal mechanical characteristics.

It is found that the first three resonant frequencies of the structure show a decreasing trend as the braided angle of the 3D braided composites increases, whereas the voltage output is slightly increased when the braided angle decreases; the first three resonant frequencies of the device decrease as the mass of the mass block B continues to increase, and the first three resonant frequencies of the device decrease as the length of the cross beam continues to increase.

When analyzing the L-shaped 3D BPCEH with the specific braided angle, it is shown that the output voltage, output power and resonant frequency are significantly affected by the magnitude of the external load resistance. Specifically, the output voltage and resonant frequency are positively related to the external load resistance, that is, they increase as the load resistance increases. The output power first shows an upward trend as the load resistance increases, reaches the maximum value, and then decreases as the load resistance continues to increase. In addition, under different excitation amplitudes, the resonant frequency of the system is maintained constant, and there is a linear relationship between the peak value of the output voltage and the excitation amplitude; the output power shows a quadratic growth with the increase in the excitation amplitude. It is found that the resonant frequency of the system can be effectively reduced by increasing the mass of mass block B, thereby extending the bandwidth of the frequency response while adjusting the voltage and power output. Meanwhile, the resonant frequency of the device can be effectively reduced by increasing the length of the beam, thus increasing the energy harvesting efficiency of the device.

## Figures and Tables

**Table 1:** The geometric parameters of the L-shaped 3D BPCEH.

Geometric Parameters	Parameter Symbol	Parameter Value (mm)
The length of the main beam of the L-shaped energy collector	*L*_1_	100
The length of the auxiliary beam of the L-shaped energy collector	*L*_2_	60
The thickness of the main and auxiliary beams of the L-shaped energy collector	*H*	1
The width of the main and auxiliary beams of the L-shaped energy collector	*b*	10
The thickness of the piezoelectric layer	*h_p_*	0.2
The thickness of three-dimensional braided composite material layers	*h_s_*	0.6
The length of the mass block A	*L_m_*_1_	5
The thickness of the mass block A	*H* *_m_*_1_	5
The width of the mass block A	*b_m_*_1_	12
The length of the mass block B	*L_m_*_2_	5
The thickness of the mass block B	*H_m_*_2_	5
The width of the mass block B	*b_m_*_2_	12

**Table 2:** The material parameters of PZT-5H.

Density *ρ* (kg/m^3^)	Elastic Modulus *E* (GPa)	Poisson’s Ratio *υ*	Piezoelectric Stress Constant *e*_31_ (C/m^2^)	Dielectric Constant *ε*_33_ (nF/m)
7500	60.6	0.289	−16.6	25.55

**Table 3:** The material parameters of glass fiber-S and TDE-85.

Material	*E*_11_ (GPa)	*E*_22_ (GPa)	*E*_33_ (GPa)	*E*_44_ (GPa)	*E*_55_ (GPa)	*E*_66_ (GPa)	*υ*_12_	*υ*_13_	*υ*_23_	*ρ* (kg/m^3^)
Glass fiber-S	85	36	36	36	15	15	0.22	0.17	0.17	2500
TDE-85	4.5	4.5	4.5	4.5	4.5	4.5	0.34	0.34	0.34	1206

**Table 4:** Periodic boundary conditions.

Surface	*x*+	*x*−	*y*+	*y*−	*z*+	*z*−
Displacement	*u*	0	0	0	0	0
	0	0	*v*	0	0	0
	0	0	0	0	*w*	0
	0	0	*w*	0	*v*	0
	*w*	0	0	0	*u*	0
	*v*	0	*u*	0	0	0

**Table 5:** The material characteristics of the composite material formed by 3D braiding technology.

Braided Angles	*E*_11_ (GPa)	*E*_22_ (GPa)	*E*_33_ (GPa)	*E*_44_ (GPa)	*E*_55_ (GPa)	*E*_66_ (GPa)	*υ*_12_	*υ*_13_	*υ*_23_
20°	28.49	11.61	11.21	4.24	4.27	2.07	0.336	0.325	0.268
25°	25.87	12.59	11.32	4.54	4.48	2.26	0.351	0.326	0.273
30°	21.73	14.37	11.36	4.86	4.68	2.57	0.334	0.329	0.278
35°	20.94	14.99	13.92	5.23	5.14	3.15	0.381	0.325	0.285
40°	18.59	15.34	14.87	5.86	5.95	3.27	0.376	0.369	0.279

**Table 6:** The first three resonant frequencies of L-shaped 3D BPCEH under different braided angles.

Braided Angles	First-Order Resonant Frequency (Hz)	Second-Order Resonant Frequency (Hz)	Third-Order Resonant Frequency (Hz)
20°	33.194	105.81	332.32
25°	32.98	105.35	331.9
30°	32.678	105.08	329.88
35°	32.619	104.79	329.52
40°	32.498	104.55	328.72

**Table 7:** The first three resonant frequencies of L-shaped 3D BPCEH under different beam lengths.

Beam Length (mm)	First-Order Resonant Frequency (Hz)	Second-Order Resonant Frequency (Hz)	Third-Order Resonant Frequency (Hz)
30	88.752	303.81	843.88
40	77.432	227.78	809.84
60	57.739	155.58	729.17
80	42.982	123.23	473.36
100	32.648	104.64	328.96

## Data Availability

The raw data supporting the conclusions of this article will be made available by the authors on request.
